# Probiotics (*Lactobacillus rhamnosus R0011* and *acidophilus R0052*) Reduce the Expression of Toll-Like Receptor 4 in Mice with Alcoholic Liver Disease

**DOI:** 10.1371/journal.pone.0117451

**Published:** 2015-02-18

**Authors:** Meegun Hong, Seung Woo Kim, Sang Hak Han, Dong Joon Kim, Ki Tae Suk, Yeon Soo Kim, Myong Jo Kim, Moon Young Kim, Soon Koo Baik, Young Lim Ham

**Affiliations:** 1 Department of Internal Medicine, Hallym University College of Medicine, Chuncheon, South Korea; 2 Department of Biomedical science, Hallym University College of Medicine, Chuncheon, South Korea; 3 Department of Pathology, Hallym University College of Medicine, Chuncheon, South Korea; 4 Department of Medicine, Columbia University, New York, New York, United States of America; 5 College of Agriculture and Life Science, Kangwon National University, Chuncheon, South Korea; 6 Department of Internal Medicine, Yonsei University Wonju College of Medicine, Wonju, South Korea; 7 Department of Emergency medical technology, Daewon University College, Jecheon, South Korea; Yonsei University College of Medicine, KOREA, REPUBLIC OF

## Abstract

**Objective:**

The role of lipopolysaccharide (LPS) and toll-like receptor 4 (TLR 4) in the pathogenesis of alcoholic liver disease (ALD) has been widely established. We evaluated the biological effects of probiotics (*Lactobacillus rhamnosus R0011* and *acidophilus R0052*), KRG (Korea red ginseng), and urushiol (*Rhus verniciflua* Stokes) on ALD, including their effects on normal and high-fat diet in mice.

**Methods:**

One hundred C57BL/6 mice were classified into normal (N) and high-fat diet (H) groups. Each group was divided into 5 sub-groups: control, alcohol, alcohol+probiotics, alcohol+KRG, and alcohol+urushiol. A liver function test, histology, electron-microscopy, interleukin (IL)-1β, tumor necrosis factor (TNF)-α, IL-6, and IL-10, and TLR 4 were evaluated and compared.

**Results:**

In the N group, probiotics, KRG, and urushiol significantly reduced levels of TNF-α (12.3±5.1, 13.4±3.9, and 12.1±4.3 vs. 27.9±15.2 pg/mL) and IL-1β (108.4±39.4, 75.0±51.0, and 101.1±26.8 vs. 162.4±37.5 pg/mL), which were increased by alcohol. Alcohol-induced TLR 4 expression was reduced by probiotics and urushiol (0.7±0.2, and 0.8±0.1 vs. 1.0±0.3, p<0.001). In the H group, IL-10 was significantly increased by probiotics and KRG, compared with alcohol (25.3±15.6 and 20.4±6.2 vs. 7.6±5.6 pg/mL) and TLR 4 expression was reduced by probiotics (0.8±0.2 vs. 1.0±0.3, p = 0.007).

**Conclusions:**

Alcohol-induced TLR 4 expression was down-regulated by probiotics in the normal and high-fat diet groups. Probiotics, KRG, and urushiol might be effective in the treatment of ALD by regulating the gut-liver axis.

## Introduction

Globally, alcohol consumption ranks third among the risk factors for disease and disability. It causes 2.5 million deaths annually, constituting 4% of all deaths worldwide [1]. Alcoholic liver disease (ALD), including alcoholic fatty liver, alcoholic hepatitis, liver cirrhosis, and hepatocellular carcinoma, is responsible for 25% of deaths due to alcohol consumption [2], highlighting the importance of ALD in the general population.

The role of the lipopolysaccharide (LPS) of the gut bacteria has been widely demonstrated in the pathogenesis of ALD [3]. Bacterial translocation from disruption of the gut-barrier function by alcohol induces endotoxemia [4]. LPS induces the expression of toll-like receptor 4 (TLR 4) in Kupffer cells by binding to the LPS binding protein and to TLR 4 with its ‘co-receptor cluster of differentiation 14’ (CD 14) and myeloid differentiation factor-2. Eventually, Kupffer cells produce pro-inflammatory cytokines, such as tumor necrosis factor-α (TNF-α) and interleukin (IL)-1β [5,6].

Saturated fatty acids, derived from animal sources, do not have double bonds between individual carbon atoms in the fatty acid chain and can promote lipotoxicity *via* inflammatory pathways [7]. However, recent data has revealed that saturated fatty acids-diet might protect against ethanol-induced liver damage [8]. Most patients with ALD are malnourished, hence saturated fatty acids or nutritional therapy might show improvement in ethanol-induced liver damage [9].

The effects of probiotics and prebiotics on ALD have been examined in many studies [10]. Probiotics composed of both *Lactobacillus rhamnosus R0011* and *L*. *acidophilus R0052*. *Lactobacillus* species are Gram-positive facultative anaerobic, microaerophilic, and rod-shaped bacteria that can suppress the growth of a broad range of Gram-negative bacteria [11]. Studies on *Lactobacillus*-treated mice with ALD have revealed a positive effect on hepatic inflammation, gut-derived endotoxemia, levels of hepatic oleic acid, and the protective gut barrier. *Lactobacillus* in particular, elicited an anti-inflammatory response and down-regulated the expression of pro-inflammatory cytokines [12,13]. It was hypothesized that probiotics could disturb the mechanisms of ALD and down-regulate the expression of pro-inflammatory cytokines.

Ginseng, the root of *Panax ginseng C*.*A*. *Meyer*, is one of the most consumed oriental herbal as both, food and medicine, for over 2,000 years. Korean red ginseng (KRG) has an immunological effect by modulating the antioxidant activity of natural killer cells. Adjuvant KRG administration improves lipid profiles, as well as the symptoms of non-alcoholic fatty liver disease. Ginsenosides have a protective effect on ethanol-induced liver injury [14,15]. Urushiol is a major organic component of the sap of the lacquer tree (*Rhus verniciflua* Stokes), and anti-inflammatory, anti-microbial, and anti-oxidative effects [16]. However, the effectiveness of urushiol for ALD has yet to be determined.

Collectively, the findings of these previous studies suggest that probiotics, KRG, and urushiol may be promising therapeutics for the treatment of ALD due to their anti-inflammatory and anti-oxidative properties and other mechanisms. In the present study, we evaluated the biological effects of probiotics, KRG, and urushiol in a mouse model of ALD, including their effects on normal and high-fat diet.

## Materials and Method

### Ethics Statement

The animals received humane care, and all procedures were conducted in accordance with the National Institutes of Health Guidelines for the Care and Use of Laboratory Animals. All procedures were approved by the Hallym University College of Medicine Institutional Animal Care and Use Committee (2011–57; 2012–27).

### Chemicals

Probiotics (a bacterial culture of L. rhamnosus R0011 and L. acidophilus R0052, 20 mg; Pharmbio Korea, Chungbuk, Korea) was stored at 4°C until use. KRG was provided as an undiluted solution by The Korean Society of Ginseng and Korea Ginseng Corp (Seoul, Korea). The provided KRG contained 7 glycosides, known as ginsenosides (mg/g): Rg1 (2.481), Rb1 (5.481), Rg3(s) (0.197), Re (2.975), Rc (2.248), Rb2 (2.175), Rb (0.566), and a moisture content of (36.68%) [17].

Sap (40 mL) from the lacquer tree was diluted to a volume of 1 L by the addition of distilled water, and subsequently extracted with 1 L of *n*-hexane twice. The hexane extract was concentrated under reduced pressure to yield brownish oil (26.9 g), which was then purified by silica gel column chromatography (Merck 7734) and eluted with 20% acetone/hexane. It was further purified by the same method (Merck 9385), followed by octadecyl silica gel column chromatography (YMC GEL ODS-A) using a gradient of dimethyl sulfoxide (Sigma-Aldrich, St Louis, MO, USA), to generate urushiol. Two types of urushiol were used: monomer urushiol and the 2–4 polymer urushiol [18].

### Animals

**Fig 1:**
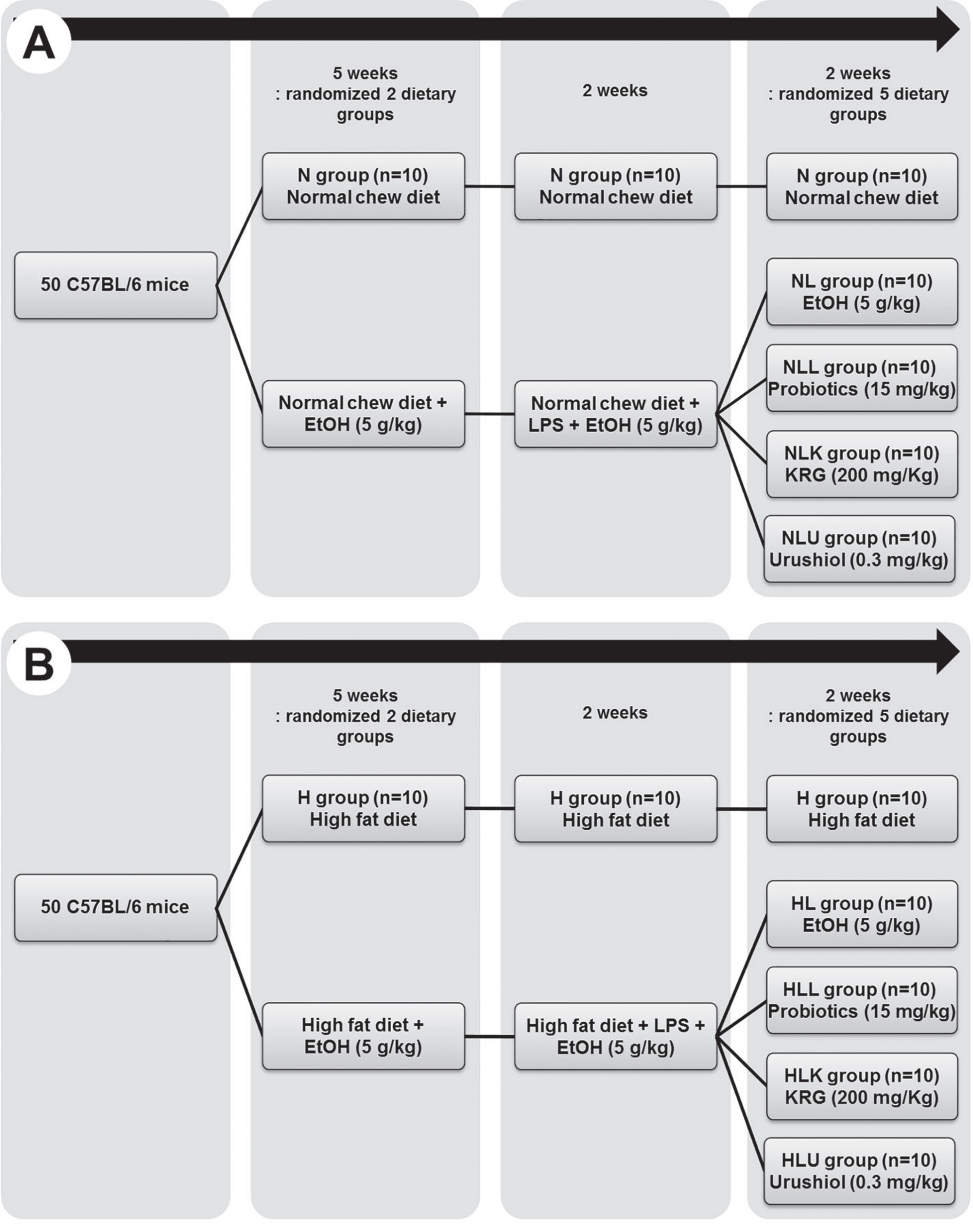
Flow chart of the study design (A) normal chew diet groups and (B) high fat diet groups. n, number; EtOH, ethanol; LPS, lipopolysaccharide; KRG, Korea red ginseng.

Age-matched (4-week-old), male C57BL/6 mice (Dooyeol Biotech, Seoul, Korea) were used in all experiments. A total of 100 C57BL/6 mice were housed individually in steel microisolator cages at 22°C with a 12/12-h light/dark cycle. All procedures were performed according to the schedule shown in Fig. 1. The 100 C57BL/6 mice were divided into normal diet (N, n = 50; 18% calories from fat, 2016; Harlan Laboratories, Indianapolis, IN, USA) and high fat diet (H, n = 50; 60% calories from saturated fat, TD 06414; Harlan Laboratories) groups. Each group was then classified equally into 5 sub-groups: (1) N group: normal chow diet for 9 weeks. (2) NL group: normal chow diet and intra-gastric ethanol for 5 weeks (5 g/kg/day, 40% ethanol) followed by intra-peritoneal injection of LPS (3 mg/kg/day, 3 times/week; derived from *Escherichia coli* serotype O55:B5 Sigma-Aldrich) + intra-gastric ethanol (5 g/kg/day twice/week, 40% ethanol) for 2 weeks, and then intra-gastric ethanol for 2 weeks (5 g/kg/day, 40% ethanol). (3) NLL group: same method for NL group with intra-gastric probiotics for last 2 weeks (1 mg/mL/day). (4) NLK group: same method for NL group with intra-gastric KRG for last 2 weeks (200 mg/kg/day). (5) NLU group: same method for NL group with intra-gastric urushiol for last 2 weeks (0.128 mg/mL/day). In H group, we gave high fat diet with the same method for N groups.

Probiotics and KRG were suspended in distilled water, while the urushiol was suspended in dimethyl sulfoxide; all 3 suspensions were administered orally using a gastric tube 5 times per week for 2 weeks. The animals were sacrificed by inhalation anesthesia overdose (isoflurane, Aerane; Baxter, Deerfield, IL, USA) at the end of the treatment periods. They were weighed, and blood, liver, and small intestine were collected. Whole blood (800 μL) samples were centrifuged (19,000 × *g* for 5 min) to collect serum. Livers were rapidly excised and stored at -80°C.

### Liver function test

Serum levels of aspartate aminotransferase (AST), alanine aminotransferase (ALT), and gamma-glutamyl transferase (γ-GT) were analyzed using a biochemical blood analyzer (Konelab 20, Thermo Fisher Scientific, Waltham, Finland). All procedures were performed by a single investigator (E.J.K) at the Center for Efficacy Assessment and Development of Functional Foods and Drugs.

### Pathology

The liver (including fatty liver) was fixed in 10% formalin and embedded in paraffin using routine methods; the tissues were sectioned at 10 μm, and sections were processed for hematoxylin and eosin (H&E), Masson’s trichrome, and reticulin fiber staining. Histopathological inflammation was classified on a 4-point scale (0–3) in accordance with the hepatitis activity index [19]. This scoring system uses a 4-point scale for inflammatory grade (grade 0: none, grade 1: minimal, grade 2: mild, grade 3: moderate). All specimens were analyzed by one hepatopathologist (S.H.H) who was blinded to the experimental conditions.

### Immunohistochemistry

Sections of formalin-fixed, paraffin wax-embedded liver tissue were stained using a Benchmark XT autostainer (Ventana Medical System, Tucson, AZ, USA) by initial immersion in pH 8.0 EDTA (ethylenediaminetetraacetic acid) antigen-retrieval buffer, followed by blocking of endogenous biotin using a biotin blocking kit (LSAB kit, Dako Canada, Mississauga, ON, Canada). Using antibodies was cluster of differentiation 45 (CD 45; dilution 1:500, Abcam, Cambridge, MA, USA), and TNF-α (dilution 1:600, Abcam). The sections were counterstained with Harris hematoxylin. The proportion of TNF-α-immunopositive areas was graded as follows: 0, <5%, 1, 5–33%, 2, 34–66%, and 3, > 66%. CD45 status was calculated as the mean of the 10 vessels using a CD 45 antigen counter (UTHSCSA ImageTool 3.0, The University of Texas Health Science Center at San Antonio, San Antonio, TX, USA). All of these analyses were also performed by S.H.H.

### Transmission electron microscopy

Changes in the tight junctions and microvilli of the small intestine were evaluated using transmission electron microscopy (TEM). The jejunum samples in the 10 groups were separated and fixed immediately with 50% glutaraldehyde and 8% paraformaldehyde, postfixed with 2% osmium tetroxide, and embedded in resin (EM-bed 812, Araldite 502, DDSA DMR 30, Electron Microscopy Sciences, Hatfield, PA, USA). Ultrathin sections were cut and stained with uranyl acetate and lead citrate. The samples were examined using a transmission electron microscope (EM 109, Zeiss, Jena, Germany) and analyzed with the aid of an electron microscope image analyzer (iTEM, Olympus, Tokyo, Japan).

### Cytokines

Serum expression of pro-inflammatory cytokines including TNF-α, IL-1β, and IL-6, and the anti-inflammatory cytokine IL-10 were analyzed by enzyme-linked immunosorbent assay (ELISA; Bio-Plex Pro Mouse Cytokine Assay kit, Bio-Rad Laboratories, Seoul, Korea) according to the manufacturer’s instructions. Lyophilized standards were supplied with the ELISA kit, and were reconstituted and diluted at 7 serial concentrations, also following the manufacturer’s protocol (standard curves). Bead fluorescence readings were achieved using Bio-Plex Manager and Luminex xPONENT software (Luminex, Austin, TX, USA).

### Western blots

Liver samples were homogenized in a complete Bio-Plex cell lysis kit (Bio-Rad Laboratories, Seoul, Korea). Proteins were then separated by SDS polyacrylamide gel electrophoresis and transferred to and immobilized on a nitrocellulose membrane, and used antibodies TLR 4 (1:500, Santa Cruz Biotechnology, Santa Cruz, CA, USA), GAPDH (dilution 1:250, Santa Cruz Biotechnology) and horseradish peroxidase (HRP)-conjugated (dilution 1:2000, 60R-AG002hrp, Fitzgerald Industries International, Acton, MA, USA). The membrane was reacted with the enhanced chemiluminescence (ECL) substrate solution (Power-Opti ECL, Bionote, KyungKi-do, Korea) and exposed to a ChemiDoc XRS+ imaging system (Bio-Rad Laboratories, Seoul, Korea).

### Statistical analysis

Continuous variables were expressed as means and standard deviations. One-way ANOVA, the Kruskal-Wallis test, Dunn’s multiple comparison test, and Tukey’s multiple comparison were performed for the body weight, liver function tests, cytokines, CD 45, and TLR 4. The χ^2^ test was performed for stage of hepatitis. A *p* value < 0.05 was considered to indicate statistical significance. All statistical analyses were performed by SPSS software (ver. 18, SPSS Inc., Chicago, IL, USA).

## Results

### Body weight

Alcohol intake generally caused a reduction in the body weight of mice; however, there was no statistical significance. Treatment with probiotics, KRG, and urushiol generally increased body weight, compared with the alcohol-treated groups. The H groups weighed more than the N groups. The H, HLL, HLK, and HLU groups appeared to gain more weight than the HL groups. However, the differences were not statistically significant (Table 1).

**Table 1:** Body weight.

	Control	Alcohol	Alcohol+Probiotics	Alcohol+KRG	Alcohol+Urushiol
Normal diet	25.6 ± 1.6	24.6 ± 1.7	28.0 ± 3.4*	28.2 ± 2.8*	30.0 ± 3.1*
High-fat diet	35.7 ± 4.4	32.1 ± 3.3	33.6 ± 3.4	33.9 ± 3.3	32.0 ± 4.5

n, number; KRG, Korea red ginseng.

* *p <* 0.05 *vs*. alcohol group

### Liver function tests

Serum levels of AST and γ-GT in the N groups were not significantly different between the NL group and other groups. The serum levels of ALT were higher in the NL group than the N group; however, the difference was not statistically significant. The probiotics, KRG, and urushiol-treated groups had significantly lower ALT levels than the NL group (Table 2). There was no difference in AST or γ-GT levels in the H groups. However, the ALT level was decreased by probiotics and KRG. The HLL and HLK groups showed lower ALT levels than the H and HL groups (Table 2).

**Table 2:** Liver function test.

Normal diet groups					
U/L mean±SD	Control	Alcohol	Alcohol+Probiotics	Alcohol+KRG	Alcohol+Urushiol
AST	43.2 ± 30.3	58.3 ± 18.3	76.7 ± 47.7	35.4 ± 34.0	41.0 ± 56.3
ALT	131.1 ± 54.5	180.9 ± 90.4	112.9 ± 49.6*	111.0 ± 49.2*	87.7 ± 45.6*
γ-GT	18.9 ± 5.2	17.8 ± 6.6	18.2 ± 4.3	20.4 ± 6.8	19.3 ± 5.5
High fat diet groups					
U/L mean±SD	Control	Alcohol	Alcohol+Probiotics	Alcohol+KRG	Alcohol+Urushiol
AST	22.3 ± 11.7	28.5 ± 12.5	14.2 ± 9.3	16.8 ± 12.5	19.7 ± 9.3
ALT	129.0 ± 43.0	145.7 ± 39.4	88.0 ± 27.3*	85.5 ± 41.3*	131.8 ± 33.8
γ-GT	17.2 ± 4.7	20.0 ± 9.2	19.9 ± 4.5	22.9 ± 5.7	16.1 ± 5.0

n, number; SD, standard deviation; AST, aspartate aminotransferase; ALT, alanine aminotransferase; γ-GT, gamma-glutamyl transferase; KRG, Korea red ginseng.

* *p <* 0.05 *vs*. alcohol group

### Pathological findings

**Fig 2:**
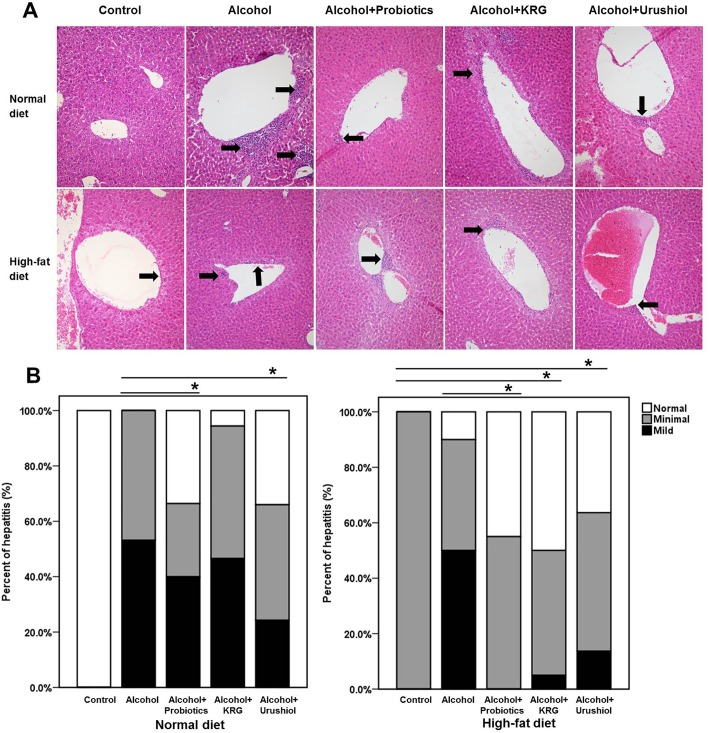
Microscope findings of liver (A; H&E, ×200) and hepatitis activity garde (B). Mononuclear cells, favoring lymphocytes, are identified in the perivenular area (black arrow). Inflammatory cells are mildly decreased in the probiotics, KRG, and urushiol groups. KRG, Korea red ginseng. * *p* < 0.05.

The NLL (*p* = 0.029) and NLU (*p* = 0.036) groups of the N groups showed improved hepatitis activity compared with the NL group. However, there was no significant difference between the NLK group (*p* = 0.632) and the NL group. Furthermore, the hepatitis activity was significantly worse in the NL, NLL, NLK, and NLU groups than in the N group (*p* < 0.001; Fig. 2).

Hepatitis was worsened by alcohol in the H groups. The hepatitis activity of the HLL (*p* = 0.020), HLK (*p* = 0.009) and HLU (*p* = 0.016) groups was less severe than that of the HL group (Fig. 2).

### Immunohistochemistry

**Fig 3:**
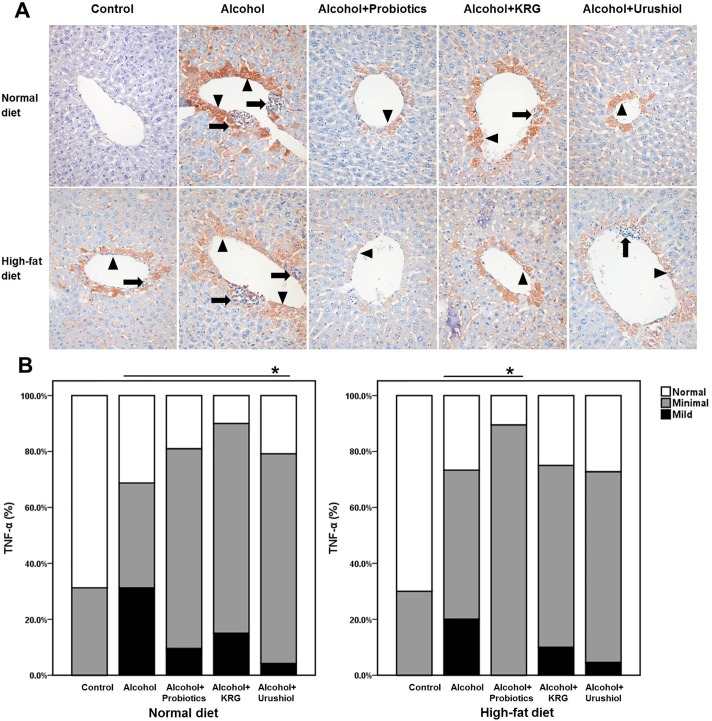
Immunohistochemical findings (A; TNF-α) and grade of TNF-α (B). TNF-α for immunohistochemical stain shows the cytoplasmic positivity (black arrowhead) in the perivenular hepatocytes around the inflammatory cells (black arrow). The intensity of the stains in the probiotics, KRG, and urushiol groups is mildly decreased than that of the alcohol only group. KRG, Korea red ginseng; TNF-α, tumor necrosis factor-α. * *p* < 0.05.

Seven and 3 mice exhibited TNF-α grade 0 and grade 1, respectively, in the N groups. Three, 4, and 3 mice showed TNF-α grades 0, 1, and 2, respectively, in the NL group. Two mice revealed TNF-α grade 0, 7 exhibited TNF-α grade 1, and 1 had TNF-α grade 2 in the NLL group. Seven mice exhibited TNF-α grade 1 and 2 mice exhibited TNF-α grade 2, in the NLK group. Two, 7, and 1 mice exhibited TNF-α grades 0, 1, and 2, respectively, in the NLU group (Fig. 3).

In the H groups, 7 mice exhibited TNF-α grade 0 and 3 mice exhibited TNF-α grade 2. In the HL group, 2 mice exhibited TNF-α grade 2. In the HLL group, 9 mice revealed TNF-α grade 1. In the HLK group, 2, 7, and 1 mice exhibited TNF-α grades 0, 1, and 2, respectively. In the HLU group, 3 and 6 mice showed TNF-α grades 0 and 1, respectively (Fig. 3).

**Fig 4:**
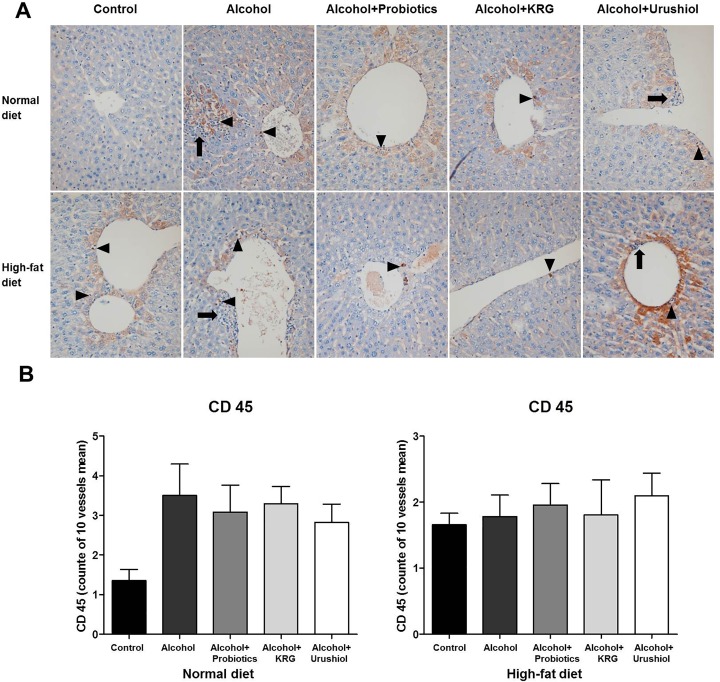
Immunohistochemical findings (A; CD 45) and grade of CD 45 (B). Mononuclear cells, favoring lymphocytes, are identified in the perivenular area (black arrowhead). The cell count is mildly decreased in the probiotics, KRG and urushiol groups. KRG, Korea red ginseng; CD 45, cluster of differentiation 45.

The CD 45 status did not differ significantly between the NL group and the other normal-chow groups (*i*.*e*., NL *vs*. N, NLL, NLK, and NLU): 3.5 ± 2.9 *vs*. 1.4 ± 1.1, 3.1 ± 3.0, 3.3 ± 1.9, and 2.8 ± 2.3, respectively; *p* > 0.05), or between the HL group and the H, HLL, HLK, and HLU groups (HL *vs*. H, HLL, HLK, and HLU): 1.8 ± 1.1 *vs*. 1.7 ± 0.5, 2.0 ± 1.2, 1.8 ± 2.4 and 2.1 ± 1.5 (*p* > 0.05; Fig. 4).

### Transmission electron microscopy

**Fig 5:**
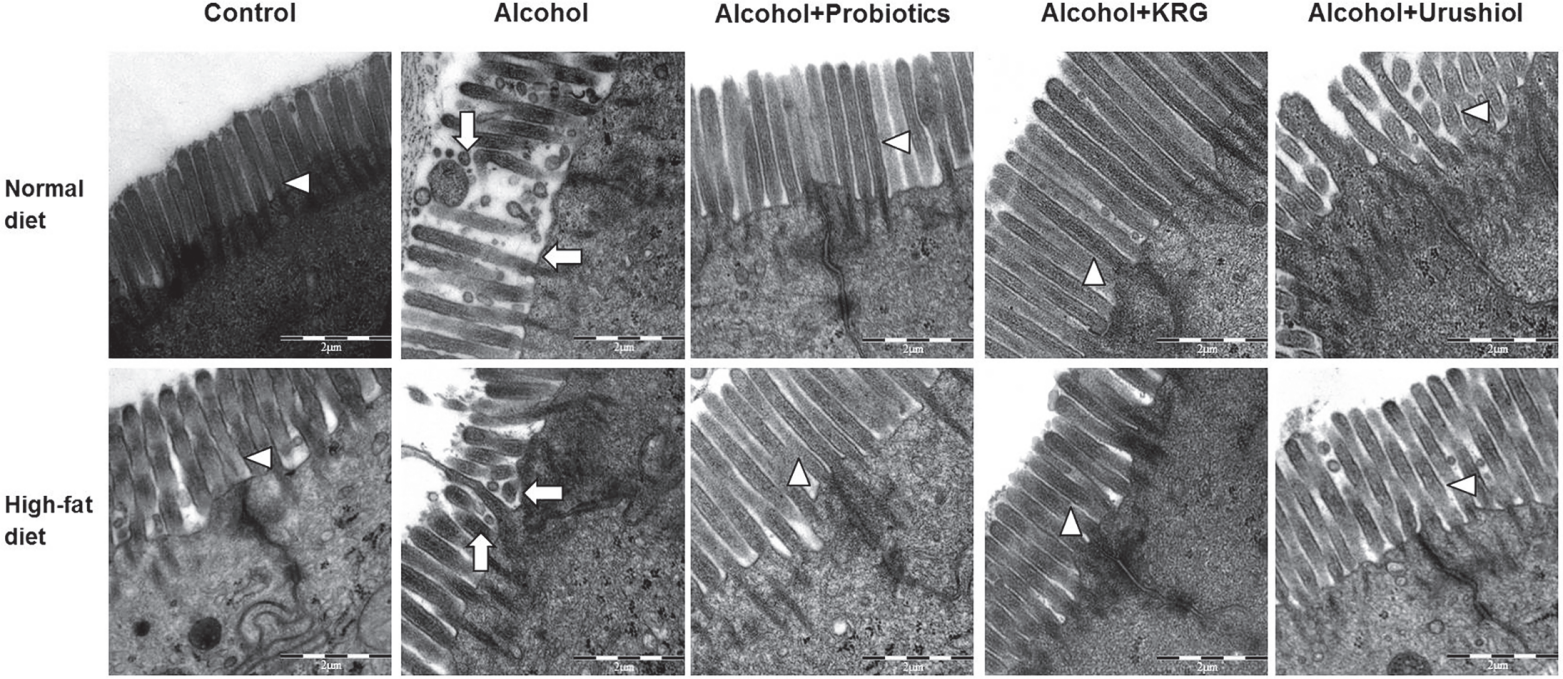
TEM findings of small intestine (×20,000). Alcohol cause irregular and deteriorated microvilli (white arrow), however, more tufted microvilli (white arrowhead) were observed in the probiotics, KRG, and urushiol groups. KRG, Korea red ginseng.

The jejunum was evaluated in 2 mice from each group, and the liver tissue was evaluated in 2 mice from each of the N, NL, H, and HL groups. Regularly arranged microvilli and undamaged tight junctions were observed in the small intestinal epithelium in the N group. Alcohol feeding appeared to cause irregular and deteriorated microvilli (NL and HL groups). However, more tufted microvilli were observed in the probiotics-, KRG-, and urushiol-treated NL and HL groups (Fig. 5).

### Cytokines

**Fig 6:**
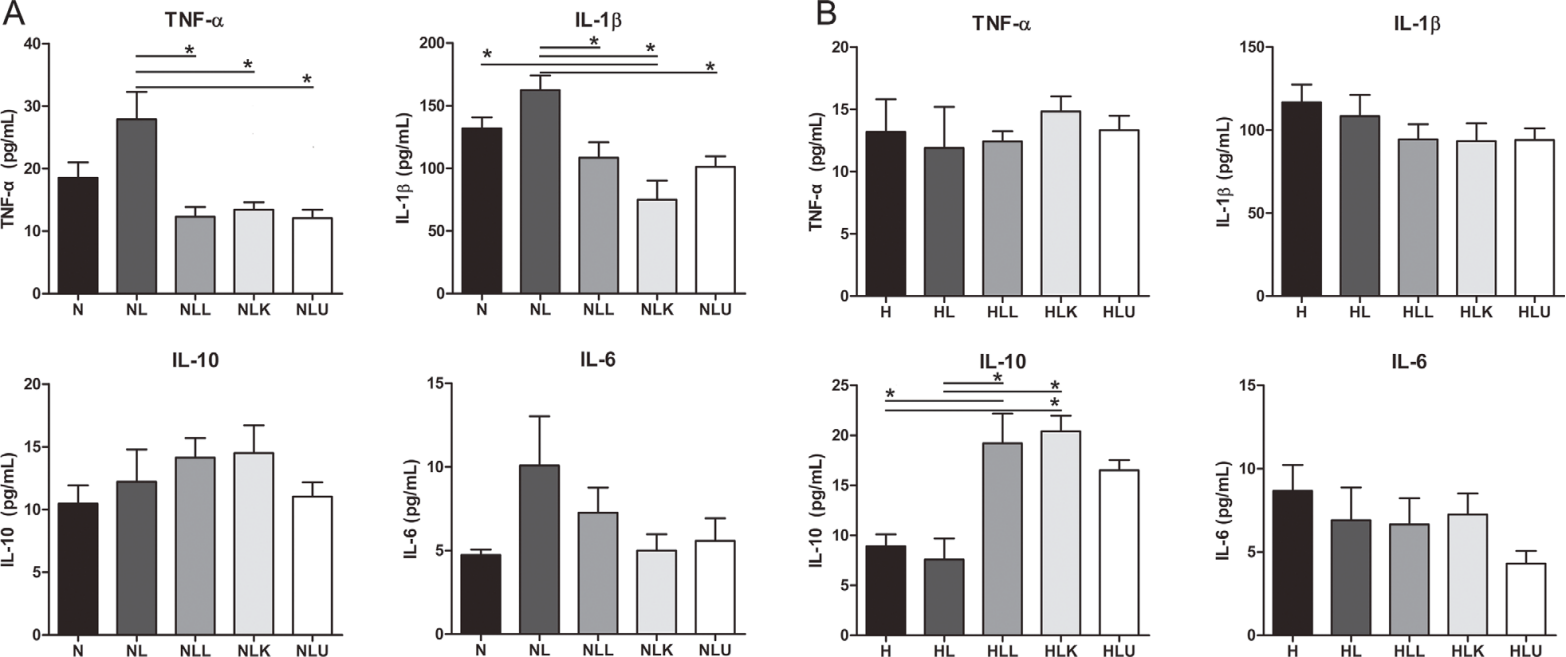
Cytokines in normal diet groups (A) and in high fat diet groups (B). Probiotics, KRG, and urushiol are effective in the reduced pro-inflammatory cytokines. IL-10 levels were significantly higher in the HLL and HLK groups than in HL. TNF-α, tumor necrosis factor-α; IL, interleukin; N, normal diet group; L, H, high fat diet; alcohol; LL, alcohol+probiotics; LK, alcohol+KRG; LU, alcohol+urushiol. * *p <* 0.05.

Cytokine analysis revealed no significant difference in the IL-10 or IL-6 levels between the NL group and the NLL, NLK, or NLU groups (*p* > 0.05). However, the levels of TNF-α in the NLL (12.3 ± 5.1 pg/mL), NLK (13.4 ± 3.9 pg/mL), and NLU (12.1 ± 4.3 pg/mL) groups were significantly lower than that in the NL group (27.9 ± 15.2 pg/mL; *p* < 0.001, *p* = 0.003, and *p* < 0.001, respectively). The IL-1β levels in the NLL (108.4 ± 39.4 pg/mL), NLK (75.0 ± 51.0 pg/mL), and NLU (101.1 ± 26.8 pg/mL) groups were also lower than that in the NL group (162.4 ± 37.5 pg/mL; *p* = 0.021, *p* < 0.001, and *p* = 0.006, respectively; Fig. 6*A*).

Cytokine analysis revealed that there was no significant difference in the TNF-α, IL-1β, or IL-6 levels between the HL group and the HLL, HLK, and HLU groups (*p* > 0.05). However, the IL-10 levels were significantly higher in the HLL (25.3 ± 15.6 pg/mL) and HLK (20.4 ± 6.2 pg/mL) groups than in H (8.9 ± 3.4 pg/mL) and HL (7.6 ± 5.6 pg/mL group) (*p* < 0.001 and *p* = 0.017, respectively). The IL-10 level was lower in the H group than in the HLL (*p* < 0.001) and HLK (*p* = 0.029) groups (Fig. 6*B*).

### Western blot

**Fig 7:**
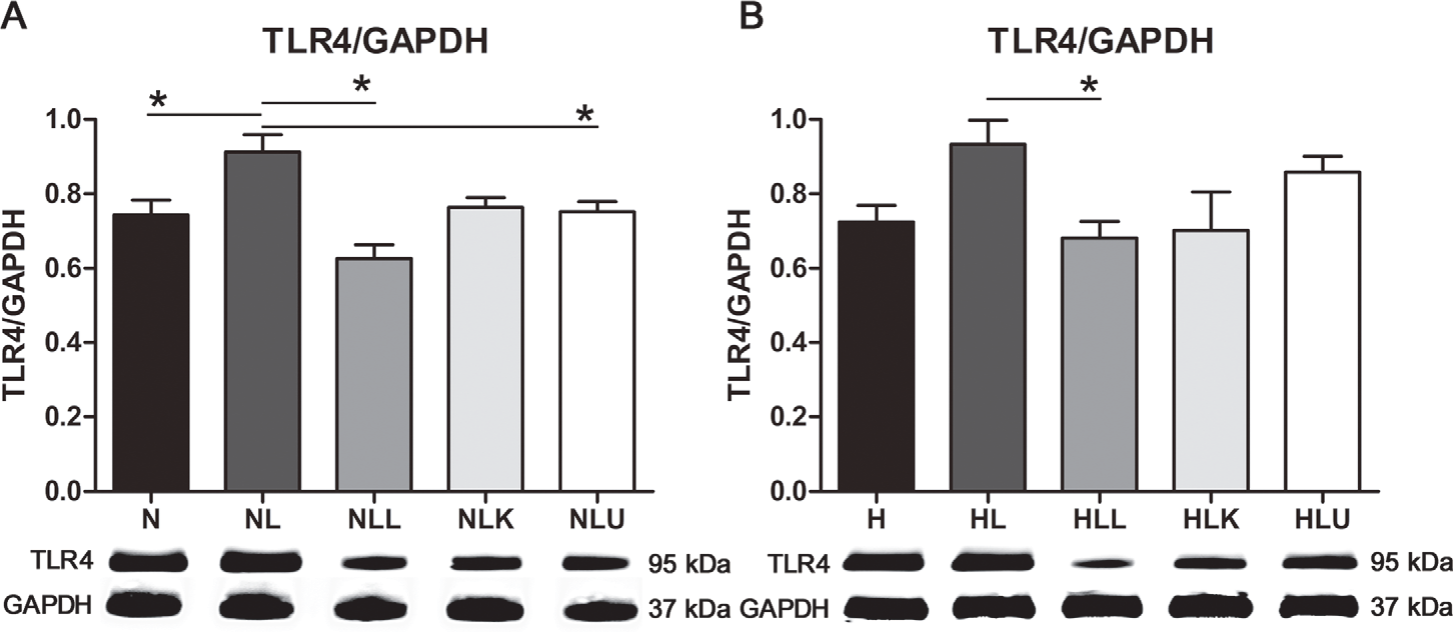
Western blot of TLR 4 in liver tissue. Alcohol induced the overexpression of TLR 4, and this was effectively down-regulated by probiotics. TLR 4, toll-like receptor 4; GAPDH, glyceraldehyde 3-phosphate dehydrogenase; normal diet group; L, H, high fat diet; alcohol; LL, alcohol+probiotics; LK, alcohol+KRG; LU, alcohol+urushiol. * *p <* 0.05.

TLR 4 was evaluated in all 10 mice in all groups. The TLR 4-to-GAPDH ratio (TLR 4/GAPDH) was significantly lower in the N (0.8 ± 0.1), NLL (0.7 ± 0.2), and NLU (0.8 ± 0.1) groups than in the NL group (1.0 ± 0.3; *p* < 0.001, *p* < 0.001, and *p* = 0.001, respectively), but did not differ significantly between the NL and NLK groups (Fig. 7*A*). TLR 4/GAPDH was significantly lower in the HLL group (0.8 ± 0.2) than in the HL group (1.0 ± 0.3, *p* = 0.017; Fig. 7*B*).

## Discussion

Alcohol induces bacterial overgrowth, especially that of Gram-negative bacteria, and translocation of LPS from the gut to the liver [6]. Moderate alcohol consumption is a strong risk factor for small intestinal bacterial overgrowth [20]. We evaluated the effect of ingested probiotics, KRG, and urushiol on the gut-liver axis in ALD. Additionally, we investigated the efficacy of a saturated fatty acid diet compared with a normal diet in ALD.

In this study, alcohol induced the overexpression of TLR 4 in liver tissue. This result was consistent with those of previous studies. TLR 4 was significantly higher in alcohol-fed rats and LPS-treated cells compared with the control conditions [21]. Another study suggested that alcohol contributed to TLR 4-mediated alcohol-induced liver injury, including steatosis, inflammation, and fibrogenesis [22]. Therefore, alcohol induced TLR 4 expression is one suggested mechanism of alcohol-induced liver injury.

Wang et al. reported that LPS-induced liver injury could be treated effectively by probiotics or steroidal anti-inflammatory drugs, and TLR 4 expression was down-regulated, regardless of the type of liver injury [23]. Another study demonstrated that lactobacilli action *via* TLR 4 represents one potential mechanism by which the increased NF-κB activity eventually reduces TLR 4 [24]. We showed that TLR 4 expression was down-regulated in response to probiotics therapy. Additionally, pro-inflammatory cytokines were reduced significantly by probiotics. As a result, it appears that probiotic therapy may be effective in the treatment of ALD by modulating the gut-liver axis.

Kim et al. found that the ginsenoside Rg5 inhibited the interaction between LPS and TLR 4 in macrophages, thus improving lung inflammation [25]. However, our results KRG did not corroborate the reported ameliorating effect. Moreover, in the present study, mice that were fed a normal diet and treated with urushiol, exhibited down-regulation of TLR 4 as compared with the alcohol-treated group. The exact mechanism of action of KRG and urushiol has not been well established to data. Further research is needed to establish the effects of KRG and urushiol in alcohol-induced models of TLR 4 expression.

The levels of pro-inflammatory cytokines such as TNF-α and IL-1β were reduced by probiotics and KRG in the normal diet groups. Other research has shown that LPS-induced inflammation can be modulated by the use of probiotics, which showed a significant effect on pro-inflammation cytokines, such as TNF-α and IL-1β in the liver and serum [26]. Some studies have demonstrated that ginsenoside Rg3 significantly attenuated pro-inflammatory cytokines (TNF-α, IL-1β, IL-6) in brain tissue induced by systemic LPS injection [27]. We can suggest that probiotics and KRG are effective in the treatment of ALD by reducing pro-inflammatory cytokines.

The present research showed that TNF-α levels in the serum and liver tissue, and IL-1β levels in the serum in the NLU group were significantly different than those in the NL groups. Urushiol affects serum cytokine (TNF-α, IL-1β) levels in *Helicobacter pylori*-infected mice [28]. Significantly decreased TNF-α and IL-1β levels were reported in LPS-treated macrophages that were administered sulfurein from *R*. *verniciflua* Stokes; while in ethanol-induced liver stellate cells, TNF-α was higher than in the normal control group [29]. As a result, urushiol ameliorates TNF-α down-regulation in both the serum and liver tissue.

The levels of the anti-inflammatory cytokine IL-10 were increased in the high-fat diet group. Mice with a saturated fatty acid diet exhibited decreased IL-10 levels in adipose tissue, while the TNF-α levels remained unchanged [30]. Previous data also showed that probiotics significantly increased the expression of IL-10 in necrotizing colitis [31]. Furthermore, IL-10 levels were increased in rats with liver fibrosis that were treated with *Panax notoginseng* saponins [32]. IL-10 is one of the most important anti-inflammatory cytokines; it inhibits TNF-α and LPS in Kupffer cells, thereby ameliorating ALD and preventing liver steatosis [33]. The current data demonstrated that in the high-fat diet groups, probiotics, KRG, and urushiol, increased the serum levels of IL-10.

Experimental models of ALD include the Lieber-DeCarli oral liquid diet, *ad libitum* oral alcohol in drinking water, Tsukamoto-French intra-gastric cannulation, the enteral feeding model, and oral gavage models [34]. Intra-gastric alcohol feeding in mice resulted in inflammatory hepatitis in 12 weeks [35]. This result was similar to our present findings. We produced ALD in mice by intra-gastric alcohol with additional intra-peritoneal LPS injection. The liver tissues of 69% of the mice showed hepatitis. One reported model used acute ethanol, followed by exposure to LPS by intra-peritoneal injection [36]. Liver damage caused by endotoxin was effectively exacerbated versus ethanol administration alone [37]. Ethanol with LPS treatment is effective as an experimental model for the development and study of ALD.

Necro-inflammation scores in the liver of the alcoholic rat model were downgraded by probiotics, and saponins from ginseng with significant pathological improvement in liver histology in ALD mice, thus preventing alcohol-induced hepatitis [38,39]. In the present study, KRG had no apparent effect on pathological findings. Probiotics therapy alone showed improvements in alcohol-induced hepatitis on histopathology. As a result, probiotics may be a therapeutic candidate for the management of alcoholic hepatitis.

In these results, alcohol-induced hepatitis score was affected by the diet. In the high fat diet group, probiotics, KRG, and urushiol reduced the grade of mild hepatitis compared with the normal diet group. Active nutritional support is known to improve biochemical markers and the nutritional status in ALD patients [40]. Consequently, sufficient nutritional support is important in the treatment of ALD.

CD 45, also called leukocyte common antigen, has increased expression in inflammatory cells with increasing severity of hepatitis and fibrosis [41]. CD 45 levels in chronic ALD patients were found to be higher than those in patients who had stopped drinking alcohol, in a clinical study [42]. Such a distinction between ALD and other groups was not made in the present research.

AST and ALT levels are one of the most representative markers of liver health and hepatotoxicity [43]. Mice on high-fat diets fed with/without alcohol did not show changes in ALT levels. Previous data indicate that *R*. *verniciflua* Stokes significantly reduced the levels of ALT in carbon tetrachloride-treated liver injury mice [44]. However, in the present study, ALT levels alone were reduced by probiotics, KRG, and urushiol. Based on the results of the present study, probiotics, KRG, and urushiol may be effective for reducing liver inflammation.

Chronic alcohol exposure can cause dysbiosis in the small intestine and increase the intestinal permeability to endotoxins such as alcohol-generated acetaldehyde; acetaldehyde can disrupt tight junctions [45,46]. In the NL group, the microvilli had deteriorated, compared with the HL group. Pretreatment of mice with probiotics, KRG, and urushiol normalized the intestinal microvilli and tight junctions compared with untreated ALD mice.

In conclusion, probiotics, KRG, and urushiol reduced levels of TNF-α and IL-1β, which were increased by alcohol in mice on a normal diet. Alcohol-induced TLR 4 expression was reduced by probiotics, and urushiol. IL-10 was increased by probiotics and KRG, compared with alcohol in the mice on a high saturated fat diet. TLR 4 was effectively down-regulated by probiotics. Thus, probiotics, KRG, and urushiol may be effective in the treatment of ALD by regulating the gut-liver axis. Probiotics may be an effective adjuvant for the treatment of ALD.

## Supporting Information

S1 Fig Western blots of TLR 4 in normal diet groups. (TIF)

S2 Fig Western blots of GAPDH in normal diet groups. (TIF)

S3 Fig Western blots of TLR 4 in high fat diet groups. (TIF)

S4 Fig Western blots of GAPDH in high fat diet groups. (TIF)
