# Supplementary figures and images for: Probiotics (Lactobacillus rhamnosus R0011 and acidophilus R0052) Reduce the Expression of Toll-Like Receptor 4 in Mice with Alcoholic Liver Disease
Source: PLoS One. 2015 Feb 18;10(2):e0117451. doi: 10.1371/journal.pone.0117451 (PMC4333821; doi:10.1371/journal.pone.0117451)

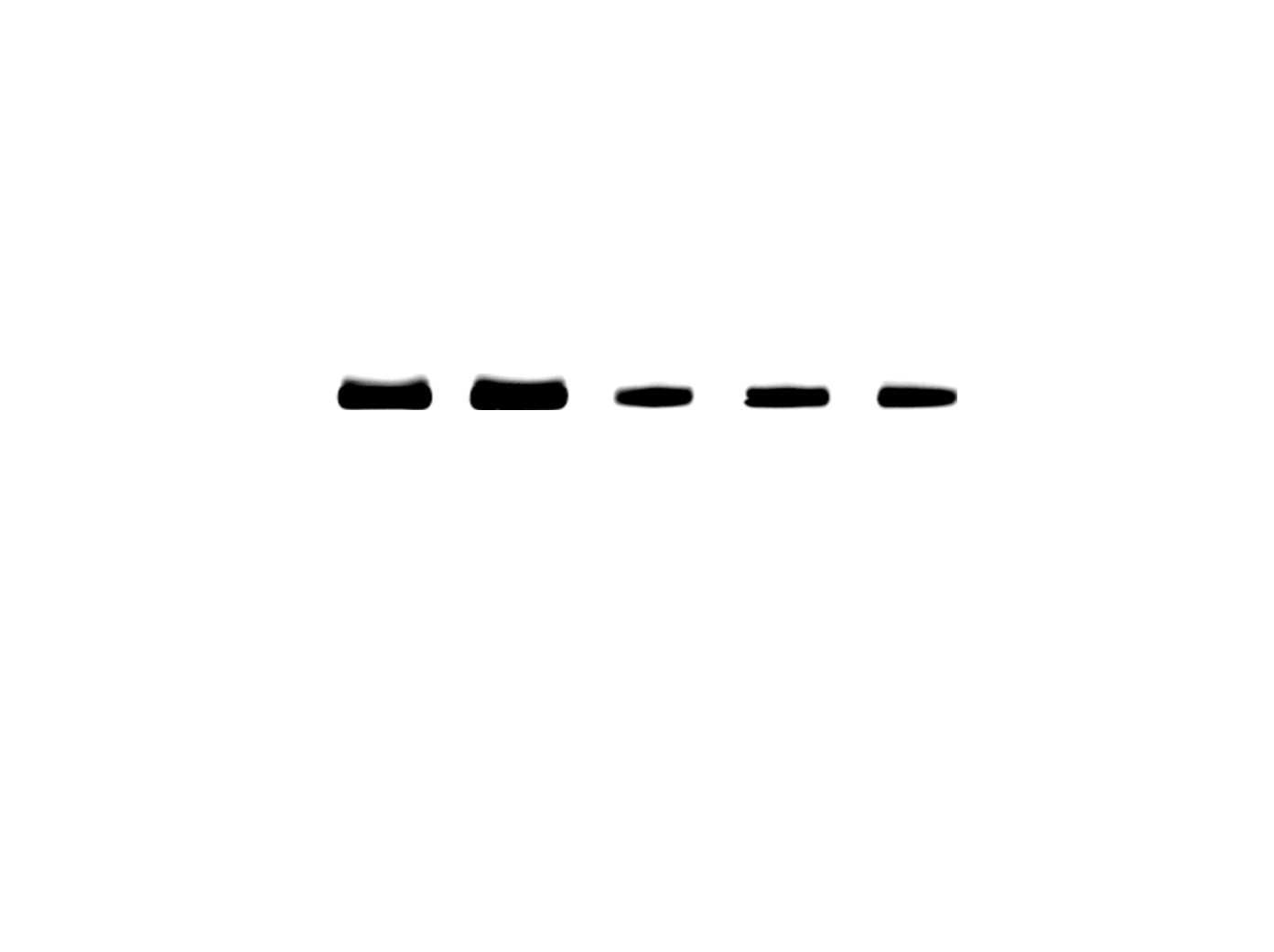

Supplement: S1 Fig — (TIF) [file pone.0117451.s001.tif]

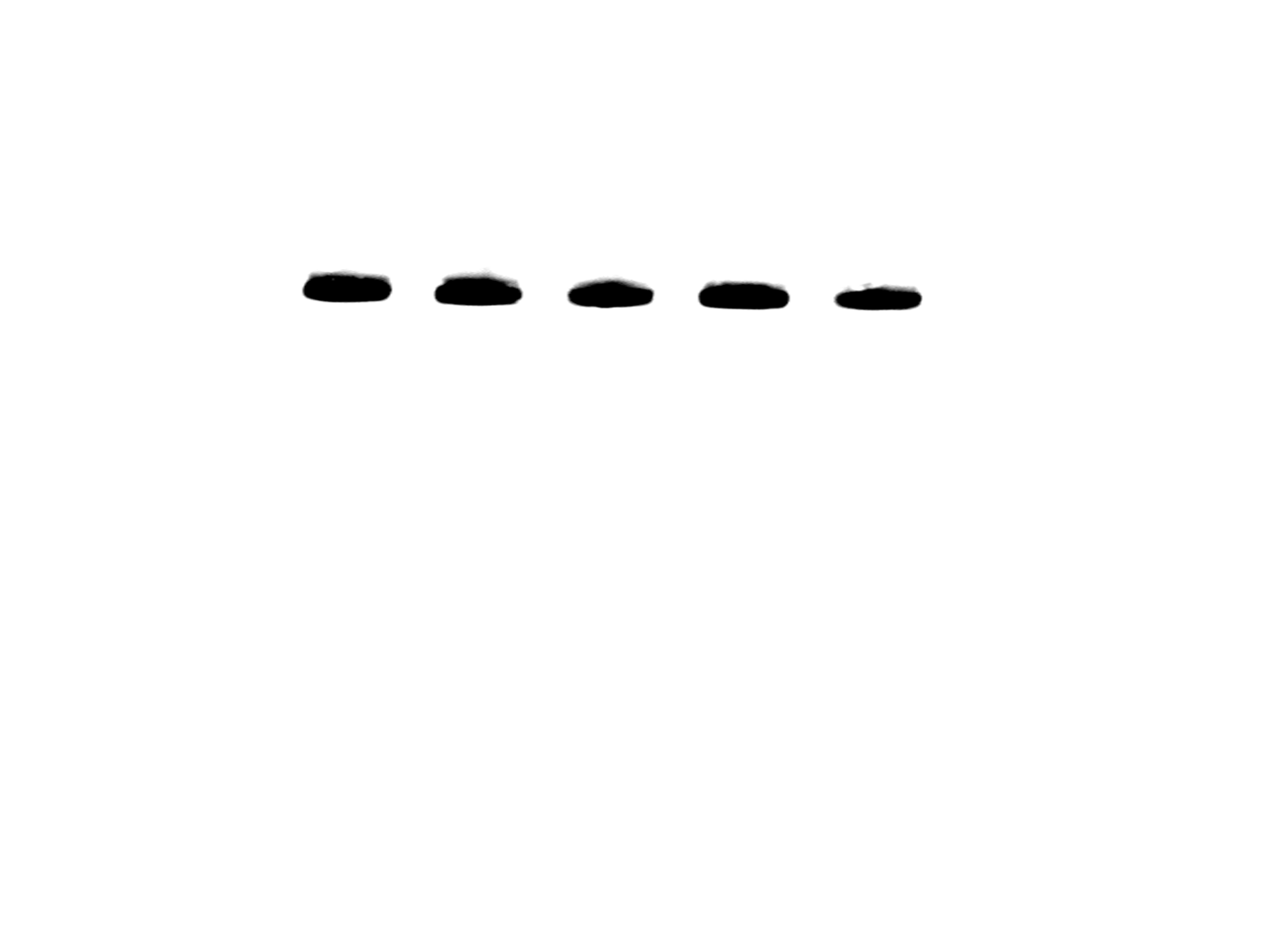

Supplement: S2 Fig — (TIF) [file pone.0117451.s002.tif]

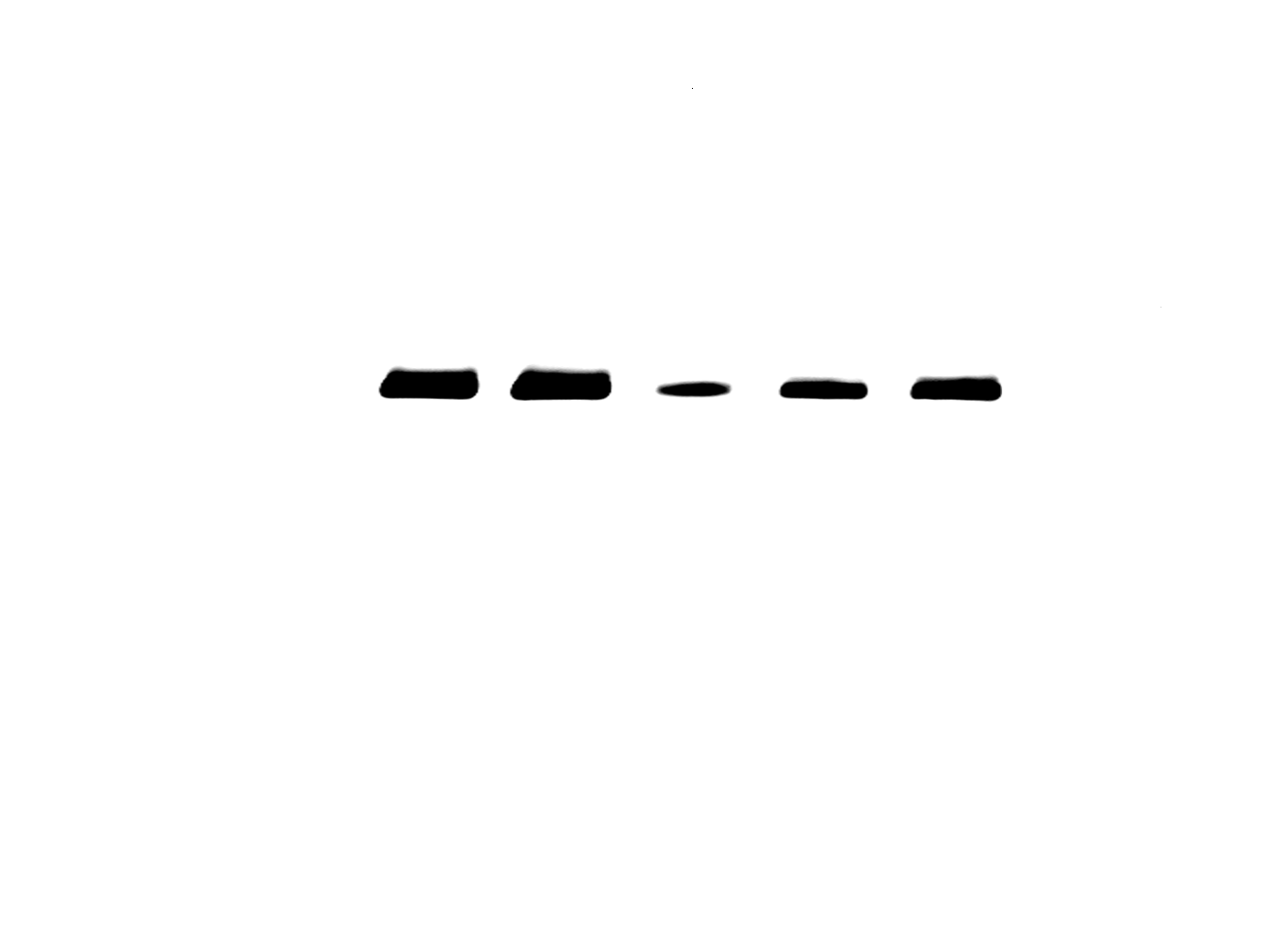

Supplement: S3 Fig — (TIF) [file pone.0117451.s003.tif]

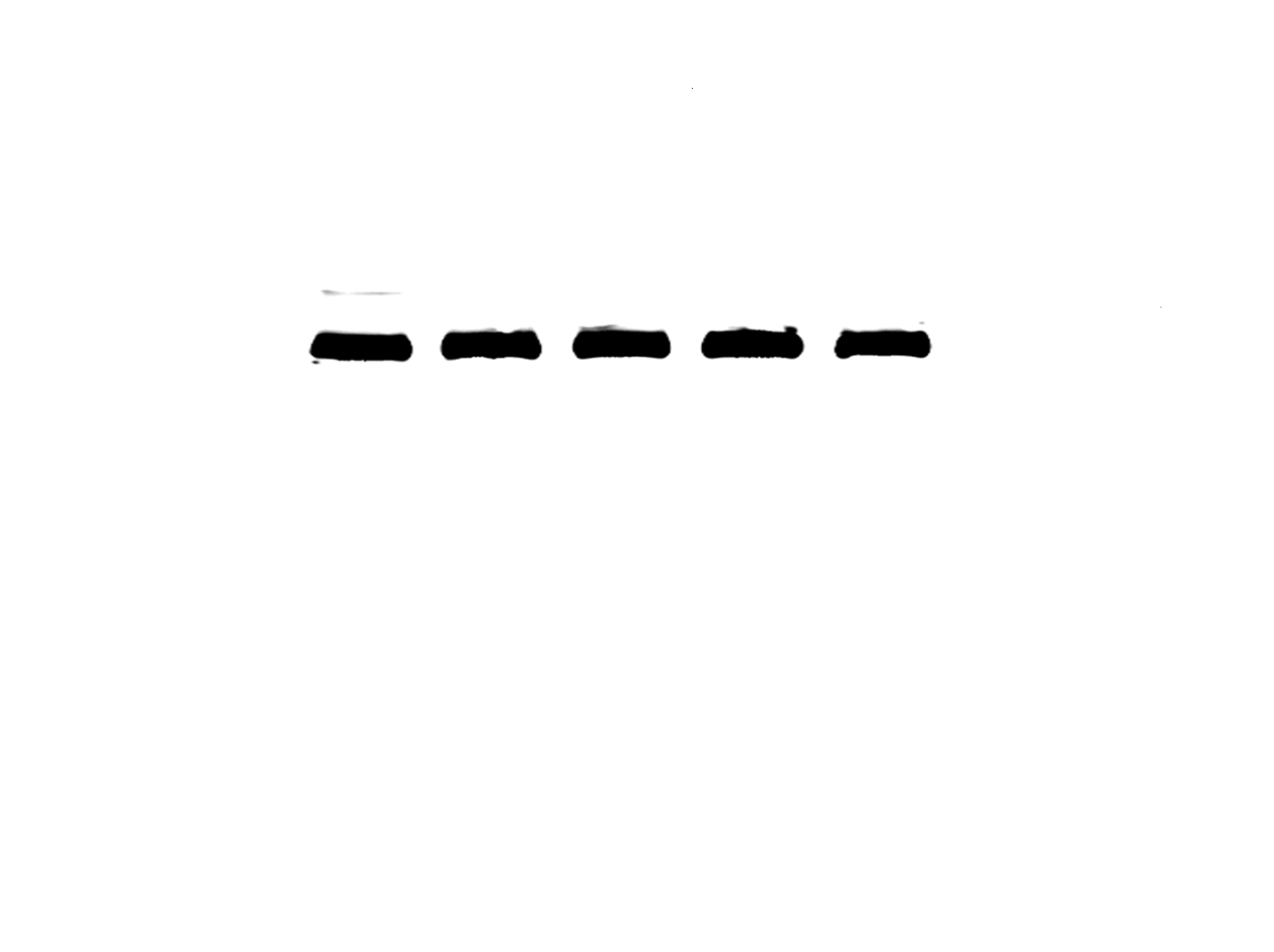

Supplement: S4 Fig — (TIF) [file pone.0117451.s004.tif]
